# Deconstruction of central line insertion guidelines based on the positive deviance approach—Reducing gaps between guidelines and implementation: A qualitative ethnographic research

**DOI:** 10.1371/journal.pone.0222608

**Published:** 2019-09-19

**Authors:** Ricky Cohen, Anat Gesser-Edelsburg, Arvind Singhal, Shmuel Benenson, Allon E. Moses

**Affiliations:** 1 School of Public Health, University of Haifa, Haifa, Israel; 2 The Health and Risk Communication Research Center, University of Haifa, Haifa, Israel; 3 Department of Communication, The University of Texas at El Paso, El Paso, Texas, United States of America; 4 School of Business and Social Sciences, Inland University of Applied Sciences, Elverum, Norway; 5 Department of Clinical Microbiology and Infectious Diseases, Hadassah-Hebrew University Medical Center, Ein-Kerem, Jerusalem, Israel; University of Maryland School of Medicine, UNITED STATES

## Abstract

**Background:**

Despite a proven association between the implementation of prevention guidelines for central line associated blood stream infections (CLABSI) and reduction in CLABSI rates, in practice there is poor adherence. Furthermore, current guidelines fail to address the multiple process on the care continuum. This research is based on the bottom-up "Positive Deviance" (PD) approach, through which multiple creative and safer solutions for central line (CL) insertion were identified that were not previously described in the guidelines. The aim of the study was to deconstruct CLABSI prevention guidelines ("during insertion" process only) through the PD approach, working with physicians to identify additional actions that, in practice, help maintain a sterile environment and contribute to patient safety.

**Methods and findings:**

Our study included a qualitative ethnographic study involving 76 physicians, working in a division of internal medicine and two intensive care units (ICUs). We triangulated findings from a combination of data-collection methods: semi-structured interviews, focused observations, video documentation, Discovery & Action Dialogue (DAD), and simulations. Deconstruction analysis was performed. A total of 23 creative extensions and variations of CL insertion practices were identified.

**Conclusions:**

The PD approach enables the identification of vital nuggets of hidden wisdom missing from the formal explicit CLABSI guidelines, and therefore helps bridge the gap between theory and praxis. During the guideline's deconstruction process, through collaborative staff learning, the written procedure is transformed into a living, breathing and cooperative one. It can reduce hospital stays and save lives, and therefore needs careful attention of healthcare scholars and practitioners.

## Introduction

Healthcare-associated infections (HAIs) acquired by patients during hospitalization are one of the most intractable and highly investigated global health problems. While HAIs are life-threating to hundreds of millions of people worldwide [1, 2], an estimated 70 percent of HAIs are preventable [3].

The European Union reports approximately 4,544,100 infections [4, 5] and 25,000 deaths [6] and the United States approximately 2,000,000 infections and 23,000 deaths annually [4, 5, 7]. A 2013 State Comptroller Report estimated the incidence of HAIs in Israel at 40,000–100,000 annually, with a mortality of 4,000–6,000 [8].

Central line associated blood stream infections (CLABSI) are the most common HAIs in intensive care units (ICUs), defined as a primary blood stream infection (BSI) in a patient who had a central line (CL) within the 48-hour period before development of the BSI, and when the BSI was not secondary to an infection at another site [9]. CLABSI's are responsible for substantial mortality, morbidity, extended length of hospital stay, and additional costs to hospitals [10, 11]. They are responsible for approximately 100,000 deaths and up to $40 billion additional annual costs to the US healthcare system, and are estimated to be the eighth leading cause of death in the US [12].

Strategies to prevent CLABSI in acute care settings were first outlined in 2008 by a group of endorsing and supporting organizations, and then updated in 2014 [13]. The latest (2017) updated Centers for Disease Control and Prevention (CDC) "Guidelines for the Prevention of Intravascular Catheter-Related Infections" was prepared by a working group comprising members from professional organizations [14, 15]. This and earlier guidance documents and guidelines were considered part of multifaceted strategies aimed at improving compliance with evidence-based recommended practices to enhance patient safety. The bundle components include the following practices: hand hygiene prior to insertion, maximal barrier precautions, chlorhexidine skin antisepsis, optimal site selection, and daily review of line necessity [16].

There is a significant association between implementation of CL insertion and maintenance bundles, and reduction in the incidence of CLABSIs in ICU settings. Nonetheless, more data is needed to determine which components of the maintenance bundle are essential in reducing infection risk [11, 13]. Ista et al.’s systematic review and meta-analysis recommended paying more attention to the implementation of CL insertion and maintenance bundles, including protocol compliance [10].

The National Healthcare Safety Network (NHSN) of the CDC has, since 2009, closely tracked US progress in reducing HAIs. The 2016 CDC progress report, based on 2014 data, indicated that CLABSI in acute care hospitals decreased 50 percent between 2008 and 2014 [17, 18]. Despite this progress and an explicit interest among ICUs to bring CLABSI rates down further, there is a poor adherence to the current guidelines on insertion and maintenance of CL. More work is needed to ensure patients’ safety when receiving medical care [18, 19].

In Israel, a decision was taken in 2014 to include CLABSI rates as part of quality of care indicators in hospitals [20]. The Israeli National Infection Control Unit’s 2018 report showed a 25 percent reduction in the average national rate of CLABSI compared to 2016, varying between 0.0 and 17.0 per 1,000 catheter days [21]. However, this is still much higher than the average rate in the US, which is less than one percent per 1000 catheter days [22].

Gurses et al. [23] analyzed the complexity of the problems physicians face in surgical ICU settings while following guidelines to prevent four types of HAIs, including CLABSIs. They found that compliance with the guidelines increased when guidelines were specific, and the roles and responsibilities of staff for performing specific tasks in their units clearly defined. Moreover, as emphasized by Gesser-Edelsburg et al. [24], the written CL insertion guidelines cannot be totally comprehensive as exigencies arise from the dynamic nature of this work, and gray areas exist in the care continuum. The guidelines focus on the temporal order of actions in their broadest sense, and these may be performed by variant staff members in different units and circumstances.

One of the creative solutions found to address these gray areas is the use of the "Positive Deviance Approach" (PD), which has been employed for nearly three decades in over 50 countries to address a wide variety of complex social problems, including the reduction of HAIs in the US, Colombia, and other countries [25–27]. The PD approach is based on the premise that in every community there are individuals or groups whose uncommon behaviors and strategies enable them to find better solutions to problems than their peers, while having access to the same resources. The PD approach is a method of grounded social inquiry in two dimensions. The first term, "Positive", refers to an action that optimizes and improves a given situation, which leads to better solutions to the same problems. The second term, "Deviance", refers to individuals who are exceptional by their uncommon behavior, which means that the majority do not take these specific actions [28].

The PD approach differs from common approaches to problem-solving, as it seeks to identify and streamline existing resources derived from the staff within a setting, rather than import external "best practices." These people and their behaviors are ordinarily “invisible” to others in their community [29]. The approach seeks to expose the solutions that are introverted over time among those people and to let the community use the same insights embodied in these practices [29].

Singhal and Svenkerud [30] proposed an alternative conceptualization of diffusion of innovations by employing the PD approach, arguing that for solving complex problems there is value in turning the classical diffusion paradigm on its head. They argue that while the classic diffusion approach favors the spread of evidence-based “best practice,” the PD approach favors the spread of practice-based evidence (i.e., the amplification of a deviant and variant practice that makes a positive difference in a given context). Thus, PD is an inside-out process, in contrast to the classical dominant framework of outside-in diffusion. Diffusion of safe CL insertion practices can be achieved by identifying positively deviant and variant practices associated with CL insertion, and then simulations can be amplified and scaled across units [21, 31, 32].

The present article seeks to deconstruct the processes associated with the guidelines for CL insertion and focuses on the insertion phase done by the physicians only, and not on the maintenance phase done by the nurses. Deconstruction is defined as a method that examines the relationship between a text and its meaning through decomposing, disassembling, and reassembling it [33]. Accordingly, in this article, we treat the CL insertion guidelines as a text, disassemble them, and then show how they can creatively be reassembled using positively deviant solutions. The positive deviants in this study are physicians, whose practices, not mentioned in the guidelines, help maintain a sterile environment in the process of the CL insertion, thus reducing the risk of CLABSIs and saving patient lives.

## Methods

### Research design

This qualitative ethnographic research method used several tools: semi-structured interviews, observations, video documentation, Discovery & Action Dialogue (DAD) [34], and simulations. Through ethnographic research undertaken in the hospitals, we identified key informants with a deeper understanding of CLABSI processes, who provided useful insights from the perspective of physicians into the process of CL insertions, and helped identify positively deviant solutions that enhanced patient safety during CL insertion [35].

The study was conducted during 2018–2019, in the General ICU (GICU), Medical ICU (MICU), and Division of Internal Medicine, of the Hadassah Ein-Kerem Hospital, Jerusalem, Israel.

### Sampling

The general research population included 76 physicians: 35 from two ICUs and 41 from the Division of Internal Medicine. During the sampling stages which were conducted in the two ICUs, we identified four physicians as PDs, as detailed below.

*In the first stage*, purposive sampling, a nonrandom technique to identify and select individuals or groups proficient and well-informed with a phenomenon of interest [36] was employed. The idea behind purposive sampling is to focus on people with characteristics relevant to our research; in our case, physicians who insert CLs. In this study we chose to focus on the physician population- the key actors in CL insertions in ICUs [37].

*In the second stage*, snowball sampling [38] was employed, a method that identifies individuals of investigative interest (e.g. the positive deviant physicians). Based on collegial recommendations, we identified physicians who demonstrated exceptional positive practices during CL insertion.

### Research tools and process

For Step 1 (data collection), we used the following tools:

1. *Interviews*: A total of 35 ICUs physicians were interviewed (Table 1). Before each interview, staff members were provided an explanation about the purpose of the study and signed an informed consent form. The semi-structured interviews were based on the DAD guidelines that had previously been used in PD work in US and Colombian hospitals [34]. The protocol included questions regarding common difficulties in maintaining hygiene during CL insertion, and any unique practices they performed during this procedure. Moreover, interviewees were asked to name staff members they believed to be PD, i.e., “Persons who demonstrated PD behaviors or who raised creative ideas in the process of CL insertion”.
2. *Observations*: We conducted 5 unobtrusive but focused observations of PD physicians who demonstrated practices related to CL insertion [38].
3. *Video documentations*: Observations and simulation were documented on video, in accordance with Bandura’s Social Cognitive Theory, which emphasizes, among other constructs, that most human behavior is influenced and learned by observing the behavior of others [39]. The videos were important for designing and developing activities to spread the PD solutions and help community members learn and practice the positively deviant behaviors that were identified during CL insertion.

**Table 1:** Sociodemographic characteristics of the ICU physicians (*n* = 35).

Characteristics	Category	*n* (%)
Gender	Male	18 (51)
	Female	17 (49)
Age (years)	Mean (SD)	36.7 (6.7)
Ethnicity	Jewish	16 (46)
	Arab	2 (6)
	Other	17 (49)
Tenure (years)	Mean (SD)	9.4 (4.8)
Department	GICU	21 (60)
	MICU	14 (40)

For Step 2 (scientific approval), data from the interviews, observations, and video documentation that informed the positive practices were presented to the Unit for Infection Prevention and Control team (two senior physicians and one nurse) for approval. We presented to them, in several sessions, the 23 practices through both written text and video clips. Initially, they discussed the practices and were asked to assess whether the PD practices were anticipated to reduce infections or cause harm, or whether there was a controversy. They then held an internal discussion and reached expert consensus. This phase was crucial for obtaining scientific approval on the implementation of the practices, before the diffusion phase.

**Fig 1:**
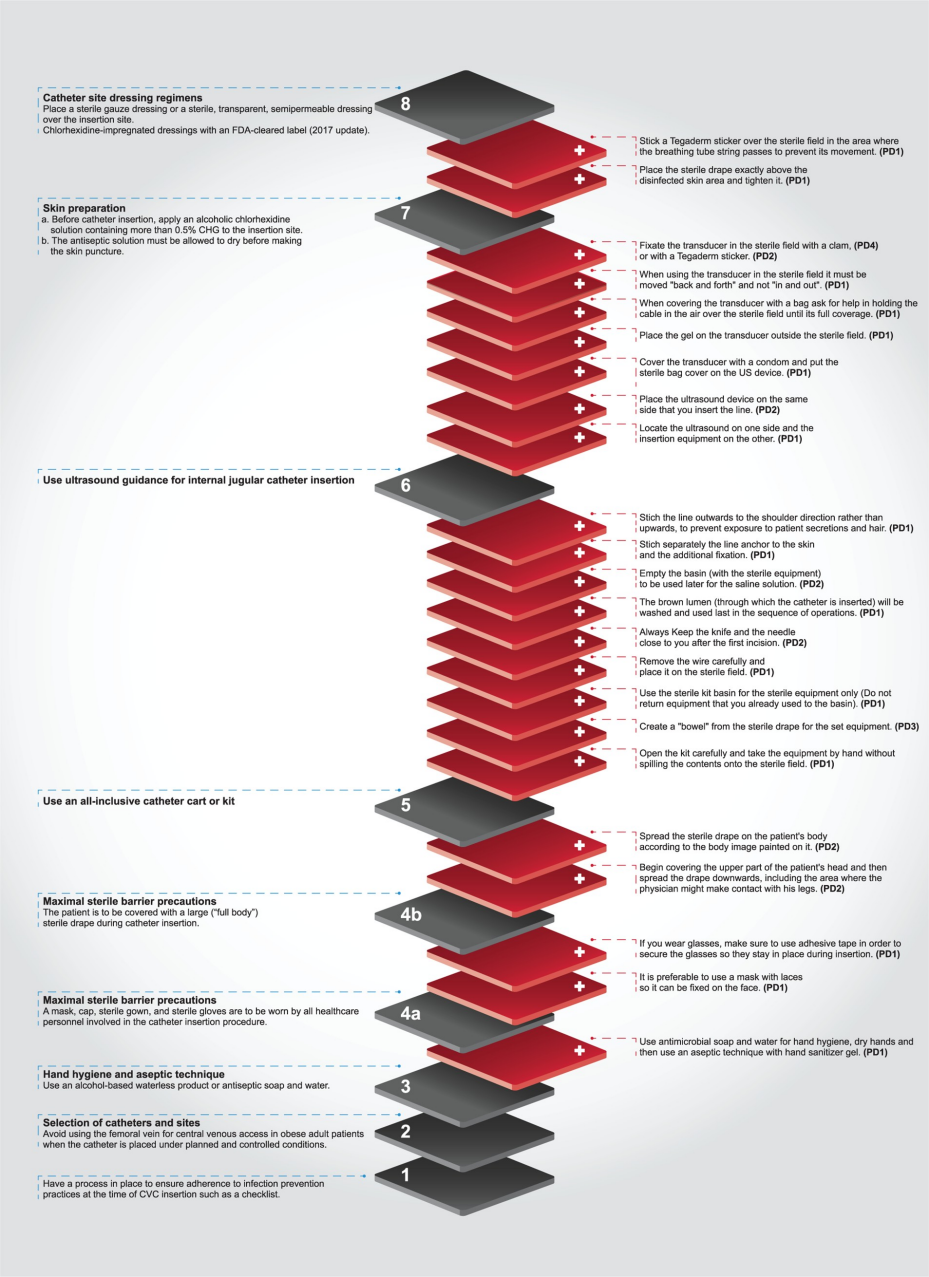
The deconstruction of CL insertion guidelines (8 orders) based on behavioral practices of physicians identified as PD (23 PD practices).

For Step 3 (diffusion of the CL insertion practices through multiple platforms) we performed the following tasks:

1. *Simulations* [31, 32]: Three hands-on learning simulations were performed by four PD physicians, all from ICUs, in order to present, step-by-step, the process of CL insertion with a reasoned explanation of the significance of the additional and variant PD practices (Fig 1) to reduce the risk for CLABSI infections. The simulations were conducted in a dedicated room at the G-ICU, with all necessary medical equipment and devices. Each simulation took up to 1.5 hours, during which the PD physicians demonstrated the temporal order of actions, highlighting the important positively deviant practices they carried out in the process of inserting the CL. The simulations were accompanied by an open dialogue in which participants were invited to ask questions and raise other ideas.
2. *Discovery & Action Dialogue (DAD) meetings*: Two meetings were held with 41staff members comprising of junior and senior physicians (10 students, 5 interns, 16 fellows, 8 specialists, 6 directors), from the Division of Internal Medicine. The recorded videos were played, questions answered, and based on the DAD guidelines, a discussion opened on the viability of disseminating these extraordinary recommended practices among other physicians [34].
3. *Editing and spreading the videos through WhatsApp application*: After collecting and videotaping all the positive practices associated with the insertion of the CL identified among the four physicians, the videos were combined into one edited video (11 minutes total length) that combined all desired practices, demonstrating all the stages for dissemination to all ICU physicians via the WhatsApp application.
4. *Israel PD conference*: Approximately 300 healthcare professionals took part in a unique professional conference dedicated to the PD approach and its implementation in Israel. The edited video of CL insertion was center stage. In addition, the PD approach, and procedures for receiving scientific approval and diffusing of PD practices were presented.
5. *PD Israel Facebook*: We opened a Facebook page entitled "Positive Deviance Israel" with the goal of creating a community and a scientific social network of public health professionals interested in the approach, and to serve as a platform for raising awareness, dissemination and exchanging information. The page contains professional content: publications, lectures, videos (including the CL insertion video), and encourages professional collaborations and engagement in the field. The page received over 1580 views in the first month (May 2019).

### Validity

We triangulated the data obtained from different sources to bolster the study’s validity: face-to-face semi-structured interviews, observations, video documentation, and simulations [40]. Interviews and observations were conducted in different day shifts for periods of several hours, and PD behaviors were documented in detail in field notes. We strengthened validity by obtaining confirmation from the Unit for Infection Prevention and Control regarding CL PD practices found.

### Data analysis

Analysis of the data was conducted as follows:

- Guidelines presentation–We integrated the three main guidelines in the literature (years: 2011, 2014, 2017) [13–15], in order to compile the 9 main categories. Those categories represent the order of actions to be taken during CL insertion (see gray layers in Fig 1).
- we integrated and categorized texts from the three guidelines (2011, 2014, 2017) into an index of “order of actions” to be taken during CL insertion [13–15].
- Content analysis and integration–we conducted a thematic analysis [41] of the texts, based on statements made by the four PD physicians during the interviews and simulations. Based on the analysis, we developed new integrated categories of the additional practices the physicians noted when performing CL insertions.
- Comparisons–we compared the CLABSI guidelines index to the additional PD practices identified.
- Deconstruction–we completed the analytical process by deconstruction, through which a common CL insertion narrative was created that contained the extension and additions to the index of current guidelines (Fig 1).

### Ethics

This research was approved by the ethics committee of The Faculty of Social Welfare and Health Sciences at the University of Haifa, Haifa, Israel (confirmation number 392/17).

## Results

A total of 23 practices were identified that were carried out on the continuum of CL insertion orders by four physicians who were identified as PD. Fig 1 provides a diagram of the various texts as they appear in the current guidelines, which shows the order of actions to be considered when inserting the CL and highlights the PDs' creative additions or expansion to current guidelines. Table 2 complements the diagram by providing PDs quotations extracted from the video simulations, where they explain the practices.

**Table 2:** Deconstruction transcripts: Description of actions during the simulations—quotes by the PDs.

The guidelines index (the order of actions for central line insertion)	Simulations & PD number	The additional behavioral practices of physicians identified as PD	Description of actions during the simulations (quotes by the PDs)
**1. Have a process in place** to ensure adherence to infection prevention practices at the time of CVC insertion such as a checklist.			
**2. Selection of catheters and sites**: avoid using the femoral vein for central venous access in obese adult patients, when the catheter is placed under planned and controlled conditions.			
**3. Hand hygiene and aseptic technique:** use an alcohol-based waterless product or antiseptic soap and water.	**Simulation 1:** PD1 **Simulation 2:** PD1	**1.** Hand hygiene with soap and water, dry and repeat with hand sanitizer gel.	*"The water from the tap is not sterile*, *so after cleaning and disinfection of the hands…alcohol must be sprayed*, *and this is something that is important to note " (PD1)*
**4a. Maximal sterile barrier precautions:** a mask, cap, sterile gown, and sterile gloves are to be worn by all healthcare personnel involved in the catheter insertion procedure.	**Simulation 1:** PD1 **Simulation 2:** PD1	**2.** Fixation devices on the face: It is preferable to use a mask with laces so that the mask can be fixed to the face. **3.** If you wear glasses, I recommend sticking them with adhesive tape so that they do not move during the insertion.	*"You need to take masks that can be tied*, *and they do not move*.* *.* *.*to those who have glasses like I do…I stick them so they will not move either" (PD1)*
**4b. Maximal sterile barrier precautions:** the patient is to be covered with a large (“full body”) sterile drape during catheter insertion.	**Simulation 3:** PD2	**4**. Begin with a cover over the upper part of the patient's head.	*"When I start to cover the patient's head with the sterile drape*, *I spread it down to the lower area of the bed*. *It also maintains sterility in the contact area with my legs when I get closer to the patient" (PD2)*
		**5**. Spread the sterile sheet on the patient's body according to the body image painted on it.	*"You might notice that starting from the head towards the legs as illustrated on the top of the kit" (PD2)*
**5. Use an all-inclusive catheter cart or kit**	**Simulation 1:** PD1	**6.** Open the kit carefully and taking the equipment by hand without spilling the contents onto the sterile field.	*"When you release (the contents of the kit) then things tend to roll out and this is a weak point…and then if one thing falls*, *what do you do*?*" (PD1)*
	**Simulation 2:** PD3	**7**. Create a "bowl" from the sterile cover for the set equipment.	*"I create a kind of bowl from the edge of the sterile cover*, *and then I release the equipment and it does not disperse" (PD3)*
	**Simulation 1:** PD1 **Simulation 2:** PD1	**8**. Use the sterile bowl for sterile equipment only (do not return equipment that you used to the bowl).	*"If I did not notice and I disqualified the needle and put it back in the bowl then I disqualified all the other equipment in the bowl" (PD1)*
	**Simulation 1:** PD1 **Simulation 2:** PD1	**9**. Remove the wire carefully to control its movement and place it on the sterile field.	*"Remove the wire carefully and note that you place it in the sterile field in case you need it again" (PD1)*
	**Simulation 3:** PD2	**10**. Always keep closer to you the skin knife and the needle after the first incision.	*"The two things you should always keep are the needle and the skin knife*.* *.* *. *because if you have to prick it again then that's what you'll need*.* *.* *. *and the other parts you've already used will be put away from the sterile field because you will not need them again" (PD2)*
	**Simulation 1:** PD1 **Simulation 2:** PD1	**11**. Pumping and washing the brown lumen (through which the catheter is inserted) will be done last in the order of operations.	*"The wire passed through the skin*, *so there is a high frequency of contamination…so either I have a syringe only for the brown lumen*, *or I start to pump blood from the extra lumens and leave the brown lumen last…*. *and when I wash the lumen with saline*, *I start again with the other lumens and wash the brown lumen at the end… that is*, *I never go from the origin of the brown lumen to the origin of the two clean lumens*.* *.* *. *and this is to prevent cross infection between them"*. *(PD1)*
	**Simulation 3:** PD2	**12**. Empty the bowl (with the sterile equipment) and leave it just for the saline.	*"I empty the bowl with the sterile equipment in one place and put the bowl with the saline (on the side of my hand)…then each time the nurse hands me a sterile syringe and I pump the saline*, *inject it to the line and throw out the syringe…and again the nurse hands me a new syringe" (PD2)*
	**Simulation 2:** PD1	**13**. Stitch the line anchor to the skin and separately stitch the additional fixation.	*"…If this construction (the line anchor) is the entry point for the skin then it will always move in and out and for that you have the extra fixation… and then if the line is drawn then the tension does not pass through it and it does not move away" (PD1)*
	**Simulation 2:** PD1	**14**. Stitch the line outward to the shoulder rather than upward, to prevent exposure to patient secretions and hair.	*"When I am stitching the line*, *I rotate it toward the shoulder and then have less contact with the head area where there is hair and secretions" (PD1)*
**6. Use ultrasound guidance for internal jugular catheter insertion**	**Simulation 1:** PD1 **Simulation 2:** PD1	**15**. Locate the ultrasound on one side and the insertion equipment on the other side.	*"Plan where you put the US device*, *where the ultrasound cable will go*, *and where you put the equipment…So that if one thing is disqualified then it will not disqualify everything" (PD1)*
	**Simulation 3:** PD2	**16**. Place the ultrasound device on the side of the hand where you insert the line.	*"The needle*, *the patient and the ultrasound are located on the same line…so I have the option to look at the same time both at the needle and at the screen" (PD2)*
	**Simulation 1:** PD1 **Simulation 2:** PD1	**17**. Put a cover bag on the US device, and double cover the transducer with a condom plus a dedicated bag that includes cable coverage.	*"You put the condom on the transducer and cover bag above it and then it's another layer of protection…" (PD1)*
	**Simulation 1:** PD1 **Simulation 2:** PD1	**18**. Place the gel on the transducer outside the sterile field.	*"It is very important when you put the gel on the transducer*, *you need to put it outside… because the particles of the gel are dispersed in any direction and can disqualify the sterile field" (PD1)*
	**Simulation 1:** PD1 **Simulation 2:** PD1	**19**. When covering the transducer with a bag ask for help in holding the cable on the air over the sterile field until its full coverage.	*"…Because what's happening*? *As soon as she (the nurse) serves you the transducer and then leaves (before you finish laying the bag) then the cable touches the sterile field…" (PD1)*
	**Simulation 1:** PD1 **Simulation 2:** PD1	**20**. Fixate the transducer on the sterile field and move it "back and forth" and not "in and out".	*"Most of the physicians I see*, *when they finish with the transducer*, *they put it aside and then the cable touches the side of the bed and it's disqualified as sterile…then what do you do when you need to use it again*?*" (PD1)*
	**Simulation 2:** PD4	**21.1**. Fixate the transducer on the sterile field with a clamp.	*"I measure the length (of the cable) at first and then wrap the cable with the sides of the sterile drape and clamp it with the clamp and then there is no possibility to move it" (PD4)*
	**Simulation 3:** PD2	**21.2.** Fixate the transducer on the sterile field with a Tegaderm.	*"The other option is sticking Tegaderm on the ultrasound cable to the sterile field" (PD2)*
**7. Skin preparation:** before catheter insertion, apply an alcoholic chlorhexidine solution containing more than 0.5% CHG to the insertion site. The antiseptic solution must be allowed to dry before making the skin puncture.	**Simulation 2:** PD1	**22**. Place the sterile drape exactly above the disinfected skin area and tighten it well.	*"…This is why the sterilizing substance is orange so that you can see exactly the disinfected skin area… Otherwise during the manipulation of the insertion*, *the ends of the line can easily enter the areas that are not tightened" (PD1)*
**8. Catheter site dressing regimens:** place a sterile gauze dressing or a sterile, transparent, semipermeable dressing over the insertion site. Chlorhexidine-impregnated dressings with an FDA-cleared label (2017 update).	**Simulation 1:** PD1 **Simulation 2:** PD1	**23**. Stick Tegaderm over the sterile field to prevent movement of the string.	*"Sometimes you have to unplug the string or move it because it is in the sterile field and it can hurt the sterility*. * *.* *. *Then when you fix it with the Tegaderm above it actually solves the problem" (PD1)*

## Discussion

The current guidelines to prevent CLABSI includes steps to be taken [13–15] without fully explaining how actions should be performed. As can be seen from the diagram (Fig 1) and accompanying table (Table 2), 23 additional detailed practices were identified by four PD physicians at the operative level and demonstrated during the study. The third category in the index sequence ("Hand hygiene and aseptic technique") begins the deconstruction chain. The findings indicate that most of the new practices were added to categories 5 (9 practices) and 6 (7 practices). In the current guidelines, category 5 does not address how to use the recommended all-inclusive catheter cart or kit, nor does it indicate the order of actions that the physician should take from the moment they open the kit until placing it on the sterile field before disqualifying it. The nine practices identified during the study describe the physicians’ step-by-step actions during the opening the kit, using the wire, washing the lumen, and about the specific location where the line is being stitched. Similarly, for category 6 ("Use ultrasound guidance for internal jugular catheter insertion"), the PD physicians (PD1, PD2, PD4) demonstrated the location of the ultrasound device, the mode of the transducer cover, the location of the ultrasound cable, and the direction of movement to be performed when using the transducer to keep the cable outside the sterile field, and thus avoiding its disqualification as being non-safe.

Since the literature shows a direct association between failure to maintain a hygienic environment and acquired infections in hospitals [3, 42], infection control guidelines play a key role in reducing HAIs [2, 13, 43, 44]. The literature reveals three major challenges in implementing the guidelines. *The first challenge* to patient safety is how to comply with the guidelines in a dynamic moment with a complex set of variables at play. The literature shows that staff members find it difficult to follow the guidelines and thus compliance rates are low [1, 45]. Other studies have sought solutions to help implement the guidelines [44], some by identifying barriers and difficulties and finding solutions to mitigate them [46]. The *second* challeng*e* is staff members’ different interpretations and implementations of the guidelines, which can often lead to failure to maintain good hygiene on the care continuum [24, 47]. The *third* challenge is the missing parts in the guidelines that we coined as "gray areas" in our previous article [24]. The grey areas are situations on the care continuum not specifically addressed in the written guidelines [24].

In this study we found that the second and third challenges to patient safety can be addressed by identifying and detailing the PD physicians’ practices to the current CL insertion guidelines. We argue that the actions of the PD physicians are deconstructionist actions of disassembly and reassembly. Accordingly, the guidelines are analogous to the text and the PD physicians are the readers. The PD physicians’ encounter with the daily reality at their hospital, generated their creative and renewed deconstructive analysis of the current guidelines. When the PD physicians attempted to perform CL insertions, they often remained with unanswered questions. Therefore, on the ground, while performing the medical procedure, they transacted an intimate and closer reading of the text, examining the texture of the guidelines and adding the creative layers that were hidden and missing from the previously explicit text. In this sense, the deconstruction and reassembly processes brought forth the hidden wisdom and made it explicit [48].

It is important to clarify in this context that our use of the word "de-construct", is not intended to refute or subvert the currentguidelines, as the philosopher Derrida explains in his writings [49]. On the contrary, the new interpretations by the PD provide an expansion, which connects and anchors the current guidelines to the ground. The PD approach corresponds with the "pragmatism" approach that uses "thought" as a tool for solving real-world problems [50, 51].

In our article the deconstruction analysis of the guidelines was done using simulation-based training [32]. The significant advantage of simulations compared to traditional learning methods is the added value they give to both the demonstrators and the audience. Simulations represent a vital step in operationalizing the PD discovery as they focus less on “telling” physicians what more to do, but instead create the enabling conditions for them to “act their way” into practicing the desired behaviors.

In our case, the simulation gave the PDs the opportunity to connect between the visualization of their thought and its rationalization. During the practical demonstration, the PDs explained to the audience their thoughts and the logic behind their actions, so that the act of inserting the CL, accompanied by words and visual cues, became an analytical process. The physician audience became active participants in the learning process, asking questions and sharing their experiences, thus significantly increasing the potential for storing and incorporating the information in their memory, rather than learning from reading literature and guidelines only [52, 53]. This process corresponds with the Cooke (2010) technique—“think-aloud protocol”, allowing the researchers to understand their thought processes and justify their actions during the simulation [54].

Kahneman and Tversky [55, 56] argued that human thought operates by two systems. The first system is an automated mode, operating at the unconscious level, and most people make decisions small and large from this mode. The second system is analytical, operating at the conscious level, and leads to the active, in-depth thinking while processing and prioritizing information. In keeping with this theory, it can be argued that during the simulations and video documentation, the act of inserting the CL, which is often performed by the medical staff in auto mode, becomes an analytical action based on in-the-moment considerations.

The dissemination of the PD practices flipped the tenets of the classical diffusion of innovation theory on its head. As Singhal and Svenkerud [30] argue, instead of just focusing on spreading evidence-based “best practices”, the PD approach turbocharges the spread of practice-based evidence (i.e., the amplification of a deviant and variant practice that makes the difference in a given context).

Disseminating the novelty of our study through different platforms (e.g. WhatsApp groups, a professional conference, and Facebook) expanded the likelihood that these analytical "human-centered” guidelines (along with the CL insertion guidelines) would disseminate to wider circles of professionals beyond the hospital where we conducted the study. In fact, this approach provides a tool aimed at encouraging physicians to find solutions to a variety of medical conditions on the care continuum that have not been addressed by the official guidelines, and thus reduces the number of "professional falls" [24]. This study seeks to encourage health centers to use this approach, which can at the same time empower staff through engagement by relaying on their "wisdom", while providing solutions through applied practices, especially in varied medical situations where there is a multiplicity of complex medical orders. Thus, the PD approach is a clarion call for increased patient safety, higher quality of care outcomes, and bringing the reservoirs of knowledge-hidden to enhance the current explicit guidelines.

Limitations of this study relate to not examining its long-term impact, that is, behavioral change and a reduction in CLABSI rates. As is known from the extensive literature in the field, significant changes such as declines in infection rates can be revealed through long-term monitoring. Nevertheless, our main goal was to examine the input rather than the impact: the way the PD physicians added new practices to the current guidelines (deconstruction). Furthermore, our study focused on CL insertion only, and did not address maintenance, which is also considered a significant strategy to prevent CLABSI and is usually within the responsibility of nurses [57]. Further studies should consider the maintenance process. Finally, we used a specific sample from only two ICUs. It is possible that further studies would spread the PD approach in other units and deconstruct additional practices.

In conclusion, the PD approach is a versatile tool that provides a solution for including the missing part from the formal explicit guidelines, and therefore helps bridge the gaps between theory and praxis. During the guideline deconstruction process through collaborative staff learning, the written procedure is transformed into a living, breathing and cooperative one. It can reduce hospital stays and save lives, and therefore needs careful attention from healthcare scholars and practitioners [25, 26, 34, 58].
